# Prognostic Role of Tumor Mutation Burden Combined With Immune Infiltrates in Skin Cutaneous Melanoma Based on Multi-Omics Analysis

**DOI:** 10.3389/fonc.2020.570654

**Published:** 2020-11-10

**Authors:** Junya Yan, Xiaowen Wu, Jiayi Yu, Yanyan Zhu, Shundong Cang

**Affiliations:** ^1^Department of Oncology, Henan Provincial People’s Hospital, Zhengzhou University People’s Hospital, Henan University People’s Hospital, Zhengzhou, China; ^2^Key Laboratory of Carcinogenesis and Translational Research (Ministry of Education/Beijing), Department of Renal Cancer and Melanoma, Peking University Cancer Hospital & Institute, Beijing, China; ^3^Key Laboratory of Carcinogenesis and Translational Research (Ministry of Education/Beijing), Department of Radiation Oncology, Peking University Cancer Hospital & Institute, Beijing, China

**Keywords:** skin cutaneous melanoma, tumor mutation burden, immune infiltrates, prognosis, The Cancer Genome Atlas

## Abstract

Tumor mutation burden (TMB) and tumor infiltrating lymphocytes have been well-recognized as molecular determinants of immunotherapeutic responsiveness in many types of cancer. However, the relationship between TMB with immune infiltrates and their prognostic role are reported occasionally in skin cutaneous melanoma (SKCM). We obtained the somatic mutation data and transcriptome profiles of 454 SKCM patients from The Cancer Genome Atlas (TCGA) database, and analyzed the mutation profiles using “maftools” package. Correlation analysis revealed that lower TMB levels conferred poor survival outcomes, associated with lower age and advanced pathological stage. Differential analysis was conducted to the genome expression between two TMB groups using “limma” package, and we identified four hub TMB-related immune genes including *CNTFR*, *CRABP2*, *GAL*, and *PAEP*. We further analyzed the underlying relationships of the copy number variations (CNVs) of four hub genes with immune infiltrates in SKCM microenvironment through TIMER database. The results indicated that diverse forms of CNVs carried by hub genes could commonly inhibit immune infiltrates. Based on the CIBERSORT method, we compared the proportions of 22 immune cells in two TMB groups and assessed their prognostic value. The data revealed that infiltrations levels of regulatory T (Treg) cell and dendritic activated cells in high-TMB group were lower than that in low-TMB group, while M1 and M2 macrophages showed the opposite trend, especially the levels of neutrophil and macrophage correlated positively with prognosis of SKCM. Finally, we constructed a TMB Prognostic Index (TMBPI) to evaluate the predictive accuracy of the four hub TMB-related immune genes. The ROC curve was drawn to assess the predictive accuracy with AUC = 0.664 and higher TMBPI conferred poor survival outcomes, which warranted further investigation and larger samples to validate.

## Introduction

Malignant melanoma is one of the most aggressive cancers, the incidence of which is rising worldwide (1–3). On the basis of the anatomical location, melanoma is subdivided into three subtypes: skin cutaneous melanoma (SKCM), acral melanoma, and mucosal melanoma (4, 5). Skin cutaneous melanoma (SKCM) is the major subtype of melanoma in Caucasians, which accounts for more than 90% (6). Nevertheless, the proportion of SKCM is approximately 20% in Asian populations (7). In recent years, advances in immunotherapy have significantly improved survival outcomes of SKCM patients. The immune checkpoint blockade (ICB) targeting PD-1/PD-L1 and CTLA-4/B7-1 have been approved for the treatment of advanced SKCM by Food and Drug Administration (FDA) (8–11). However, the overall efficacy rate of PD-1 blockade in SKCM is approximately 26 to 44% (8, 12), thus indicating more than 50% of SKCM patients are not suitable for ICB therapy. Therefore, identification and characterization of potential biomarkers and their application in combination with immunotherapy are urgently required.

In recent years, there are many well-recognized molecular determinants of immunotherapeutic responsiveness, including PD-L1 expression on tumor (13), microsatellite instability (14), tumor mutation burden (TMB) (15), neo-antigen load (16), and tumor infiltrating lymphocytes (TILs) (17). A number of clinical trials have explored the relationship between PD-L1 expression and immunotherapeutic response in several types of cancer, such as non-small cell lung cancer (NSCLC), esophageal cancer, and SKCM (18). The results of KEYNOTE-042 (NCT02220894) study indicated that NSCLC patients with PD-L1 tumor proportion score (TPS) of 1% or greater could benefit from pembrolizumab monotherapy (19). Subgroup analysis showed that NSCLC patients with PD-L1 TPS of 50% or greater have a better survival outcome compared with patients with PD-L1 TPS of 1 to 49%. The retrospective analysis revealed that positive association of PD-L1 expression levels with a better prognosis in SKCM patients treated with ICB therapy (20). TMB was calculated as (total count of variants)/(the whole length of exons), in which the detected variants included base substitutions, insertions, or deletions across bases. Many studies demonstrated that higher TMB in tumors is inclined to form more neo-antigens that make tumors harboring higher immunogenicity, and thus lead to improved clinical response to immunotherapy (21). A series of clinical trials showed that high TMB confers high response rate and sustainable response to ICB therapy in many types of cancer (22, 23).

Nevertheless, PD-L1 expression and TMB are still not perfect biomarkers for immunotherapy, as responses are observed in PD-L1 negative or low TMB patients. A pooled analysis of two trials showed that significant benefit (i.e. including complete and durable responses) with nivolumab in all PD-L1 subgroups, including PD-L1 negative NSCLC patients (24). This may be attributed to the immune status of tumor microenvironment. ICB therapy exerts anti-tumor effects depending on the involvement of TILs, thus the abundance of TILs could be regarded as one biomarker for predicting the immunotherapeutic efficacy (25). Given the limitation of single biomarker, a prognostic model containing various biomarkers may guide immunotherapy more precisely.

A recent study investigated the correlation between TMB and immune signatures in different types of cancer (26). However, a systemic exploration of the relationship between TMB with immune infiltrates in SKCM remains lacking, so we performed this study to explore the prognostic role of TMB and its potential association with immune infiltrates in SKCM.

## Materials and Methods

### Muti-Omics Data Acquisition and Processing

First, we obtained the somatic mutation data of SKCM samples from The Cancer Genome Atlas (TCGA) database using the GDC tool (http://portal.gdc.cancer.gov/). Since the raw SNP data were not publicly available, we selected the “Masked Somatic Mutation” data and processed it through VarScan software. We prepared the Mutation Annotation Format of somatic variants and implemented the “maftools” R package which provides various functions to perform most commonly used analysis in cancer genomics and to create feature-rich customizable visualizations (27). Then, we downloaded the transcriptome profiles with HTSeq-FPKM format of all available SKCM samples compared with normal tissues. Moreover, the clinical and pathological data of SKCM patients were also obtained *via* the GDC tool, including age, gender, tumor-node-metastases (TNM) stage, and follow-up with vital status. According to the American Joint Committee on Cancer classification (AJCC) on Melanoma classification, T, N, and M stage refer to tumor thickness, regional lymph nodes metastases, and distant metastases. Since all the data in this research were form public databases, there was no ethical conflict to declare.

### Calculation of TMB Scores and Prognostic Analysis

TMB was defined as the total count of coding errors of somatic genes, base substitutions, deletions, or insertions across per million bases. In this study, we calculated the mutation frequency with number of variants/the length of exons (38 million) for each ample through Perl scripts based on the JAVA platform. The estimated TMB data of SKCM samples are shown in **Table S1**. Then, we classified the SKCM samples into high-TMB and low-TMB groups according the median data. Kaplan-Meier analysis with log-rank test was subsequently performed to compare the survival difference between two groups. In addition, we further evaluated the associations of TMB levels with clinical characteristics *via* Wilcoxon rank-sum test. According to the tumor classification of malignant degree, we divided the SKCM samples into two groups with the median level.

### Differentially Expressed Genes and Functional Pathways Analysis

According to the TMB levels, we divided the transcriptome data of SKCM samples into high- and low-TMB groups *via* R software. We selected the “limma” package to conduct the differentially expressed gene (DEGs) analysis in two groups with | Fold change (FC) | >1 and False Discovery Rate (FDR) <0.05. The heatmap plot was drawn using “pheatmap” package. Then, “org.Hs.eg.db” package was used to get the Entrez ID for each DEG and we conducted the Gene ontology (GO) and Kyoto Encyclopedia of Genes and Genomes (KEGG) analysis with “clusterProfiler,” “ggplot2,” and “enrichplot” packages. Besides, gene set enrichment analysis (GSEA) software was obtained from the home website (http://software.broadinstitute.org/gsea/index.jsp ) and worked on JAVA platform. We used the TMB level as the phenotype and selected the “c2.cp.kegg.v7.0 symbols.gmt gene sets” as the reference gene set. Furthermore, we obtained a list of immune related genes from the Immunology Database and Analysis Portal (Immport) to select the differentially expressed immune genes between the two groups using “VennDiagram” package.

### Survival Analysis

We selected the top 25 immune genes with |FC| >1 and FDR <0.05 to further assess the prognostic value of differential immune genes in patients with high- and low-TMB levels. The survival Kaplan-Meier analysis was conducted *via* a “for cycle” R script to identify the hub immune genes associated with survival outcomes and P value was shown in plot. A P value <0.05 was regarded as the statistical significance.

### TIMER Database and CIBERSORT Algorithm

We further evaluated the copy number variations (CNVs) of hub immune genes with immune infiltrates in SKCM based on the “SCNA” module of TIMER database (http://cistrome.shinyapps.io/timer/) (28). The known CNV types of hub genes were shown at the right bottom. The distributions of each immune cell subset at each CNV status in SKCM were presented by box plots and the difference of infiltration level in each category *versus* normal was compared using two-sided Wilcoxon rank sum test with calculated P value.

Meanwhile, we obtained the transcriptome profiles of SKCM patients in two TMB levels and conducted the normalization process using “limma” package. Then, we put the preparation data into subsequent analysis to evaluate the immune infractions of each sample through the CIBERSORT algorithm (R script v1.03), providing an estimation of the abundances of member cell types in a mixed cell population, using gene expression data. The CIBERSORT was still based on a known reference set, providing a set of gene expression features of 22 leukocyte subtypes-LM22. The differential distributions of immune cells in two TMB levels were shown using “pheatmap” package. The Wilcoxon rank-sum test was conducted to precisely assess the differential abundances of immune infiltrates between two TMB levels, which were shown with P value using “vioplot” package.

### Prognostic Analysis of Infiltrating Immune Cells in SKCM

Based on the TIMER database, we further performed the multivariate Cox of immune infiltration cells, which was fitted by function coxph() from R package “survival.” The hazard ratio (HR) with 95% confidence interval (95% CI) was calculated. Furthermore, batch Kaplan-Meier analysis was conducted to indicate the differential survival outcomes between different levels of immune infiltrates. A P value <0.05 of log-rank test was regarded as the statistical significance.

### Construction of TMB Prognostic index (TMBPI) for Hub Immune Genes

We performed the multivariate Cox regression analysis to obtain the respective coefficients (βi) of 4 hub immune genes. As described previously (29), the TMBPI formula was defined as: TMBPI = Ʃ(βi × Expi) (i = 4). Then, we performed the Receiver Operating Characteristic (ROC) curve to evaluate the predictive value of 4 immune signature in SKCM. Moreover, Kaplan-Meier analysis was conducted to compare the survival difference in two groups, where we divided the SKCM patients into high- and low-risk groups with the median prognostic index as the threshold.

### Statistical Analysis

The Cox regression model was performed based on the “survival” package. “Limma” package was utilized to conduct the normalization and differential analysis. Wilcoxon rank-sum test was a non-parametric statistical hypothesis test mainly used for comparisons between two groups and Kruskal-Wallis test was suitable for two or more categories. All statistical analysis was implemented based on the R software (Version 3.6.3). A P value <0.05 was thought to be statistically significant.

## Results

### Patient Characteristics

Four hundred and fifty-four patients with SKCM were included in this study and their clinicopathological characteristics are listed in **Table 1**. The patients, 281 males (61.89%) and 173 females (38.11%), were aged 15 to 90 years (median age, 63 years). According to the American Joint Committee on Cancer classification (AJCC) on Cancer classification, there were 226 (49.78%) cases of stage Is and II, 192 (42.29%) cases of stages III and IV, and 36 (7.93%) cases of unknown stage.

**Table 1:** Baseline characteristics of 454 SKCM patients included in this study.

Variables	Number (%)
Vital status	
Alive	214 (47.14)
Dead	240 (52.86)
Age (year)	
≤65	292 (64.32)
>65	162 (35.68)
Gender	
Female	173 (38.11)
Male	281 (61.89)
AJCC-T	
T0	23 (5.07)
T1	42 (9.25)
T2	76 (16.74)
T3	89 (19.60)
T4	152 (33.48)
Unknown	72 (15.86)
AJCC-N	
N0	223 (49.12)
N1	73 (16.08)
N2	49 (10.79)
N3	55 (12.12)
Unknown	54 (11.89)
AJCC-M	
M0	405 (89.21)
M1	23 (5.07)
Unknown	26 (5.72)
Stage	
I & II	226 (49.78)
III & IV	192 (42.29)
Unknown	36 (7.93)

SKCM, skin cutaneous melanoma; AJCC, American Joint Committee for Cancer; T, tumor; N, regional lymph node; M, metastasis.

### Landscape of Mutation Profiles in SKCM

**Figure 1:**
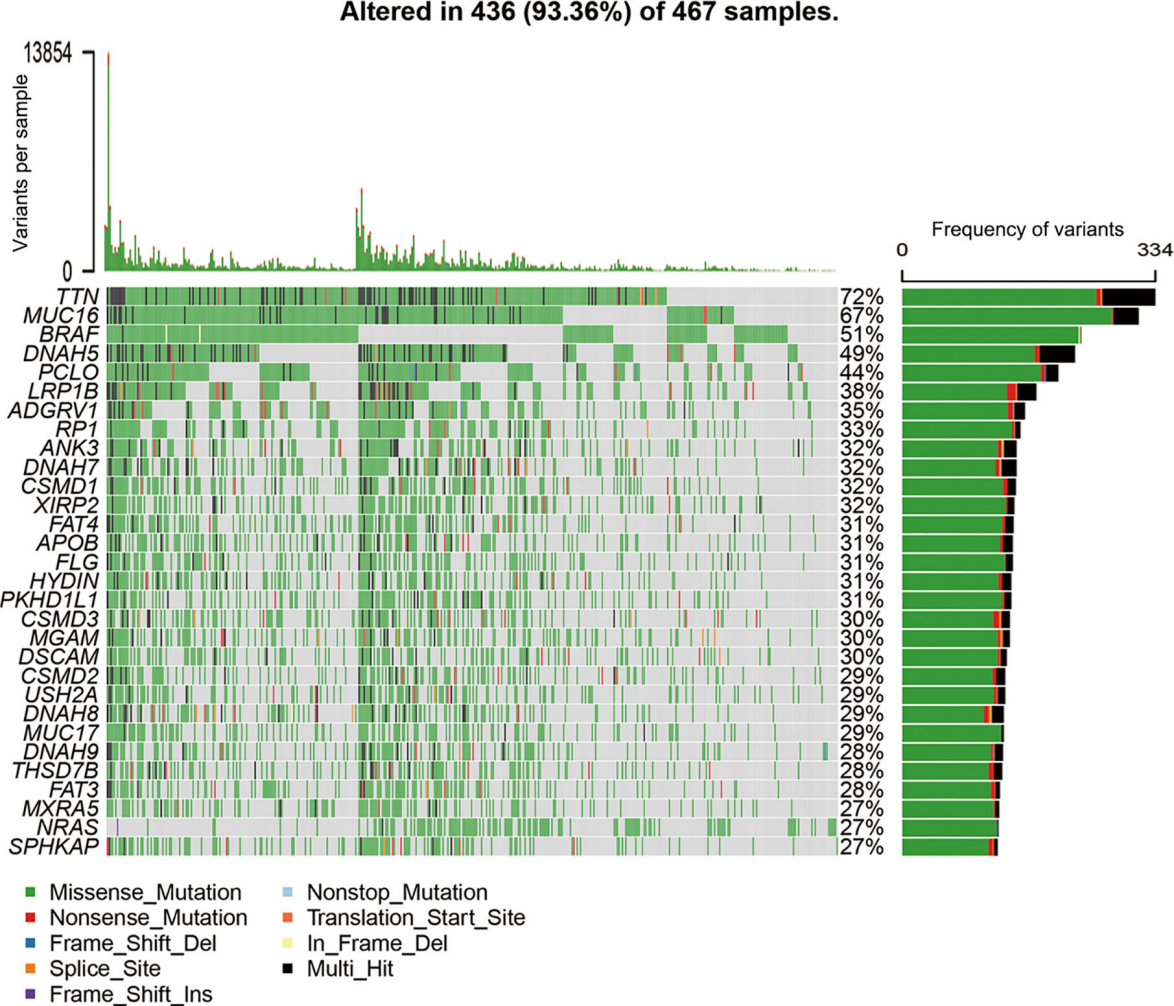
Landscape of mutation profiles in SKCM samples. The top panel showed the number of variants in each sample, and the bottle panel exhibited the mutation types of high-frequency mutated genes with various colors in each sample. In the right panel, the barplot showed the mutation frequency of each gene in all 467 samples. SKCM, skin cutaneous melanoma.

All mutation information of each gene in each sample were exhibited in waterfall plot, where various colors with annotations at the bottom represented the different mutation types (**Figure 1**). On the whole, these mutations were further summarized in different groups, in which missense mutation accounts for the most fraction (**Figure S1A**), single nucleotide polymorphism occurred more frequently than deletion or insertion (**Figure S1B**), and C>T was the most common of single nucleotide variants in SKCM (**Figure S1C****)**. Moreover, we calculated the number of altered bases for every sample and showed the mutation categories with various colors in box plots (**Figures S1D, E****)**. Horizontal histogram revealed the top 10 mutated signature in SKCM with percentages, including TTN (72%), MUC16 (67%), BRAF (51%), DNAH5 (49%), PCLO (44%), LRP1B (38%), ADGRV1 (35%), RP1 (33%), ANK3 (32%), and DNAH7 (32%) (**Figure S1F**). The coincident and exclusive associations across mutated genes were shown in **Figure S1G**, in which green represented the co-occurrence and red represented the mutually exclusive relationships. Although BRAF mutation may be exclusive to other gene mutations, the differences did not reach a significance. Meanwhile, the mutated incidences of other genes were shown in Genecloud plot (**Figure S2**). Furthermore, we evaluated the above gene variants through cBioPortal for Cancer Genomics (**Figure S3**). The results verified the reliability and accuracy of mutation profiles in SKCM.

### Correlation of TMB to Survival Outcomes and Clinicopathological Features of SKCM

**Figure 2:**
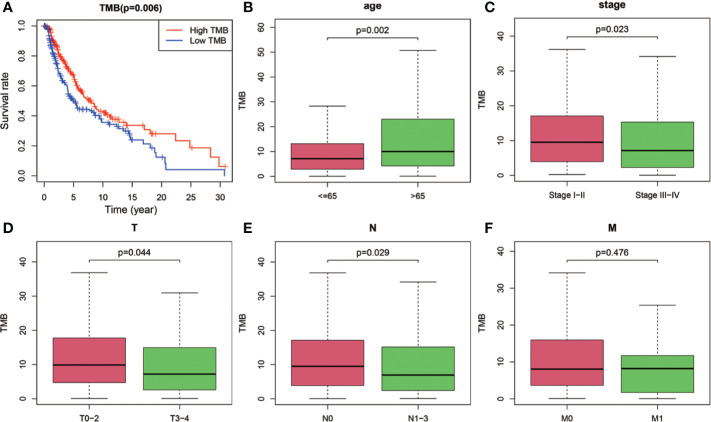
Prognostic analysis of TMB and associations with clinical risk characteristics. **(A)** Higher TMB levels correlated with better survival outcomes with P = 0.006. **(B–E)** Higher TMB level was associated with higher age, lower AJCC-T stage, lower AJCC-N stage, and early pathological stage. **(F)** No significant difference of TMB levels was observed with AJCC-M stage. TMB, tumor mutation burden; T, tumor; N, regional lymph node; M, metastasis.

After dividing TMB into two groups according to the median level, we analyzed the prognostic significance of TMB for overall survival (OS). The results indicated that SKCM patients in low-TMB group had a significantly shorter OS than that in high-TMB group (P = 0.006; **Figure 2A**). Furthermore, we examined the relationship between TMB and clinicopathological features. Statistical analysis showed that higher TMB level correlated with higher age (P = 0.002, **Figure 2B**), lower AJCC-T stage (P = 0.044, **Figure 2D**), lower AJCC-N stage (P = 0.029, **Figure 2E**), and early pathological stage (P = 0.023, **Figure 2C**). However, no significant association was found between TMB and AJCC-M stage (**Figure 2F**).

### Comparison of Gene Expression Profiles Between Two TMB Groups

**Figure 3:**
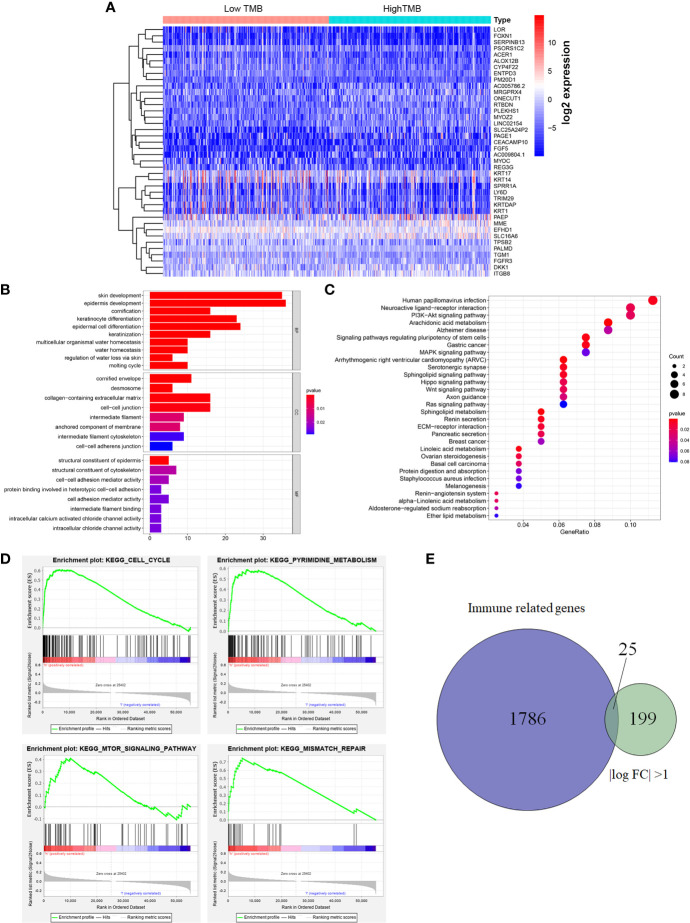
Differential analysis of gene expression profiles in high- and low-TMB groups and enrichment pathway analysis. **(A)** Heatmap of top 40 DEGs was drawn to reveal different distribution of expression state, where the colors of blue to red represented alterations from low expression to high expression. **(B, C)** GO and KEGG enriched results revealed that these differentially genes might be related to skin development, epidermis development, cell differentiation crosstalk, and multiple cancer-related crosstalk. **(D)** GSEA showed the top TMB-related crosstalk, including cell cycle, pyrimidine metabolism, mTOR signaling pathway, and mismatch repair with FDR <0.25. **(E)** TMB-related immune genes were identified through the intersection of immune related genes and DEGs between high- and low-TMB group with |log FC| >1. TMB, tumor mutation burden; DEGs, differentially expressed genes; GO, Gene Ontology; KEGG, Kyoto Encyclopedia of Genes and Genomes; GSEA, gene set enrichment analysis; FC, fold change; BP, biological process; CC, cell component; MF, molecular function.

The heatmap showed that the genome expression levels commonly decreased in high-TMB group than that in low-TMB group (**Figure 3A**). Differential analysis revealed a total of 224 DEGs with |log FC|>1 and FDR <0.05 (**Table S2**). We then conducted the GO enrichment analysis and these DEGs were mainly in skin development, epidermis development, and cell differentiation crosstalk (**Figure 3B**, **Table S3**). KEGG pathway analysis indicated that the enrichment of TMB-related signature correlated with multiple cancer-related crosstalk, such as PI3K-AKT signaling pathway, and Wnt signaling pathway (**Figure 3C**, **Table S4**). In addition, we also selected partial GSEA results of the top TMB-related items, including cell cycle, pyrimidine metabolism, mTOR signaling pathway, and mismatch repair with FDR <0.25 (**Figure 3D**). We further identified 25 immune related genes from Immport database for subsequent analysis (**Figure 3E**, **Table 2**).

**Table 2:** Differential immune genes between high-TMB and low-TMB groups.

Gene	Low group	High group	FC	logFC	P value	FDR
REG3G	0.404	2.489	0.162	−2.624	0.005	0.028
CXCL14	10.127	44.060	0.230	−2.121	<0.001	0.002
SLPI	14.781	50.258	0.294	−1.766	0.001	0.007
DEFB1	1.712	7.033	0.243	−2.039	0.006	0.031
PAEP	233.441	53.848	4.335	2.116	0.011	0.049
CRABP2	15.835	37.732	0.420	−1.253	<0.001	<0.001
PI15	5.623	11.346	0.496	−1.013	0.010	0.044
ELN	2.116	4.364	0.485	−1.044	<0.001	<0.001
PLA2G2A	3.254	7.884	0.413	−1.277	<0.001	<0.001
SYTL1	0.936	2.093	0.447	−1.162	0.002	0.014
EDN3	18.306	37.586	0.487	−1.038	<0.001	0.002
SEMA3D	1.346	2.931	0.459	−1.122	<0.001	<0.001
BMP6	1.117	2.712	0.412	−1.280	<0.001	<0.001
CSPG5	1.564	3.153	0.496	−1.012	<0.001	0.001
DKK1	6.221	3.043	2.045	1.032	0.002	0.012
FGF5	0.582	0.062	9.433	3.238	0.001	0.011
GAL	1.961	4.915	0.399	−1.326	<0.001	<0.001
IL33	2.748	5.718	0.481	−1.057	<0.001	0.002
ADCYAP1R1	0.132	0.313	0.422	−1.246	0.001	0.007
CNTFR	1.635	4.071	0.402	−1.316	<0.001	<0.001
FGFR2	0.179	0.755	0.238	−2.073	<0.001	<0.001
FGFR3	0.616	4.900	0.126	−2.992	<0.001	0.001
LGR5	0.111	0.600	0.185	−2.432	0.001	0.011
VIPR1	0.086	0.406	0.212	−2.240	<0.001	0.002
MAP3K8	1.090	2.284	0.478	−1.066	0.002	0.013

TMB, tumor mutation burden; FC, fold change; FDR, false discovery rate.

### Identification of Hub TMB-Related Immune Genes and Associations of CNVs With Immune Infiltrates in SKCM

**Figure 4:**
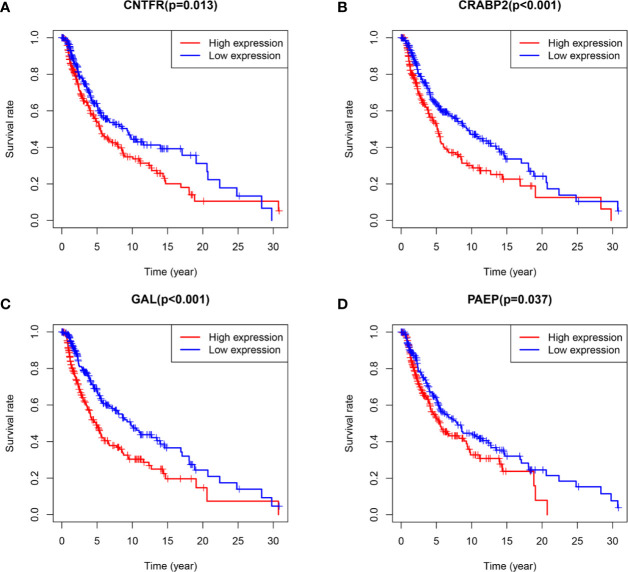
Survival analysis of four hub TMB-related signature with P value of log-rank test. **(A–D)** Higher expression levels of *CNTFR*, *CRABP2*, *GAL*, and *PAEP* correlated with poor survival outcomes, respectively. TMB, tumor mutation burden; *CNTFR*, ciliary neurotrophic factor receptor; *CRABP2*, cellular retinoic acid binding protein 2; *GAL*, galanin and GMAP prepropeptide; *PAEP*, progestogen-associated endometrial protein.

**Figure 5:**
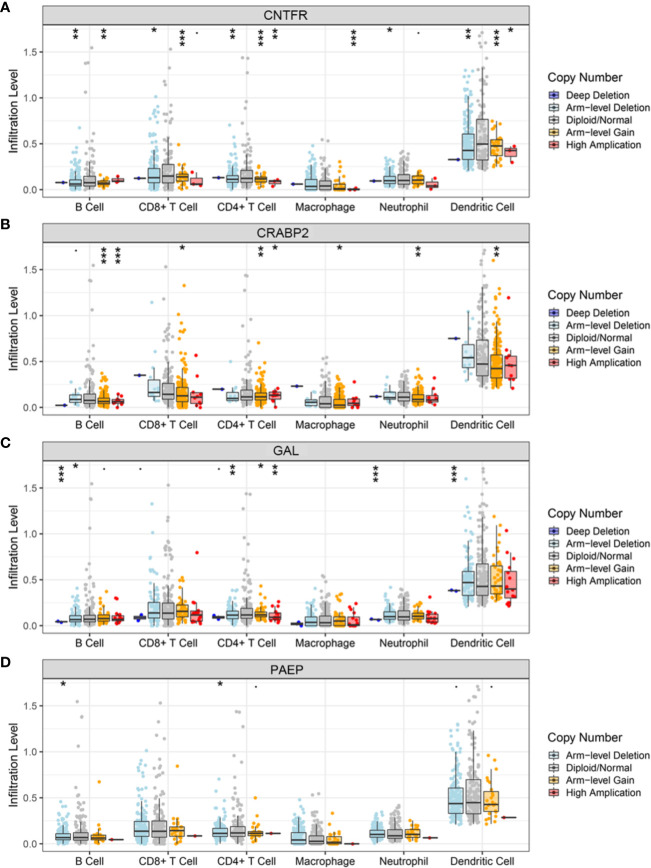
Relationships of the CNVs of four hub TMB-related genes with immune cells infiltration. **(A–D)** Immune infiltration levels of CD8+ T cell, CD4+ T Cell, neutrophil cell, dendritic cell, macrophage, and B cell were commonly decreased in diverse forms of CNVs compared with copy number normal of *CNTFR*, *CPABP2*, *GAL*, and *PAEP*. CNVs, copy number variations; TMB, tumor mutation burden; *CNTFR*, ciliary neurotrophic factor receptor; *CRABP2*, cellular retinoic acid binding protein 2; *GAL*, galanin and GMAP prepropeptide; *PAEP*, progestogen-associated endometrial protein. *, <0.05; **, <0.01; ***, <0.001.

As the workflow in **Figure S4**, batch survival analysis indicated that four prognostic hub immune genes that highly associated with survival outcomes. Higher expression levels of *CNTFR* (ciliary neurotrophic factor receptor), *CRABP2* (cellular retinoic acid binding protein 2), *GAL* (galanin and GMAP prepropeptide), and *PAEP* (progestogen-associated endometrial protein) were correlated positively with poor survival outcomes (**Figures 4A–D**). Meanwhile, the prognostic value of four hub immune genes were validated in uveal melanoma cohort from TCGA. The results showed that higher expression of *CNTFR*, *CRABP2*, and *PAEP* were correlated positively with poor survival outcomes (**Figure S5**). Moreover, we also analyzed the underlying relationship of the CNVs of four hub genes with immune infiltrates in SKCM microenvironment. Compared with the immune infiltration levels in samples with normal copy number of the signature, diverse forms of CNVs carried by four hub genes could commonly inhibit immune infiltrates, including CD8+ T cell, CD4+ T Cell, neutrophil cell, dendritic cell, macrophage, and B cell (**Figures 5A–D**).

### Differential Abundance of Immune Cells in Two TMB Groups

**Figure 6:**
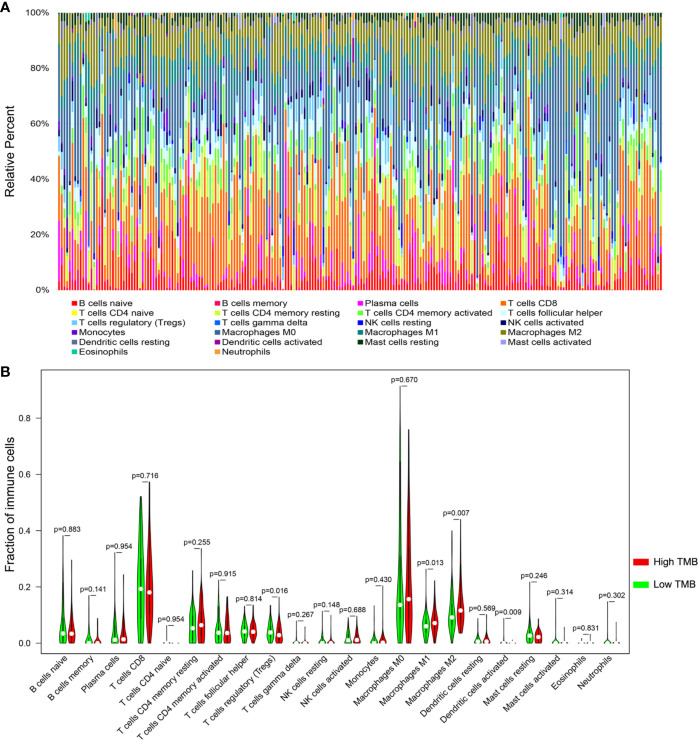
Comparisons of 22 important immune fractions between high- and low-TMB groups. **(A)** The specific 22 immune fractions represented by various colors in each sample were shown in bar plot. **(B)** Wilcoxon rank-sum test revealed that the infiltrations levels of regulatory T cell and dendritic activated cells in high-TMB group were lower than that in low-TMB group, while M1 and M2 macrophages showed the opposite trend. TMB, tumor mutation burden.

Based on the newly developed CIBERSORT method, we calculated the proportions of 22 immune cells in each SKCM sample (**Table S5**) and exhibited the result in box plot, in which various colors represented different cell subsets (**Figure 6A**). Besides, Wilcoxon rank-sum test revealed that infiltration levels of regulatory T cell and dendritic activated cell in high-TMB group were lower than that in low-TMB group, while M1 and M2 macrophages showed the opposite trend (**Figure 6B**).

### Low Neutrophil and Macrophage Infiltrates Confer Poor Survival Outcomes

**Figure 7:**
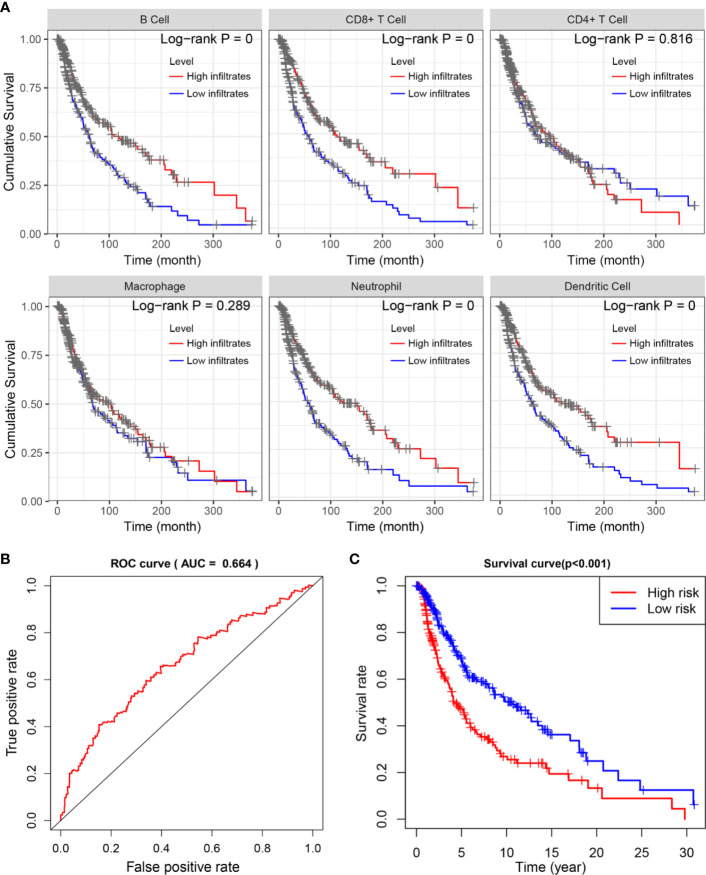
Survival analysis of differentially immune cells across two TMB groups. **(A)** Kaplan-Meier analysis revealed that lower infiltration levels of B cell, CD8+ T cell, neutrophil, and dendritic cell correlated with poor survival outcomes in SKCM. **(B, C)** Construction and assessment of TMBPI for SKCM (AUC of 3-year OS prediction = 0.664), where patients with higher TMBPI conferred poor survival outcomes (P < 0.001). TMB, tumor mutation burden; SKCM, skin cutaneous melanoma; TMBPI, Tumor Mutation Burden Prognostic Index; AUC, Area Under Curve; ROC, Receiver Operating Characteristic; OS, overall survival.

To investigate the underlying prognosis of immune cells, we performed the univariate analysis of infiltration levels of six immune cells associated with OS. The results indicated that lower infiltration levels of B cell, CD8+ T cell, neutrophil, and dendritic cell correlated with poor survival outcomes in SKCM (**Figure 7A**). Furthermore, we conducted the multivariate Cox regression model and the data showed that lower infiltration levels of macrophage and neutrophil were risk factors for SKCM (**Table 3**).

**Table 3:** Multivariate Cox regression analysis of immune infiltration cells in SKCM.

Cell types	coef	HR	95% CI	P value
B cell	−0.870	0.419	0.014–12.799	0.618
CD8+ cell	−0.737	0.479	0.046–5.009	0.539
CD4+ cell	1.891	6.624	0.353–124.366	0.206
Macrophage	2.249	9.476	1.132–79.356	0.038
Neutrophil	−6.088	0.002	0.000–1.026	0.050
Dendritic	−0.929	0.395	0.072–2.160	0.284

R square = 0.067 (max possible = 9.93e-01); Likelihood ratio test P = 5.16e-05; Wald test P = 3.72e-04; Score (logrank) test P = 3.5e-04. SKCM, skin cutaneous melanoma; HR, hazard ratio; CI, confidence interval.

### Construction and Assessment of TMBPI for SKCM

Given the relationship between alteration of immune signature with lower immune infiltrates and poor prognosis, we further evaluated the predictive accuracy of the four hub TMB-related immune genes. Based on the multivariate Cox regression analysis, we constructed the TMBPI as the following formula: TMBPI = 0.011581 × *CNTFR* + 0.006230 × *GAL* + 0.002504 × *CRABP2* + 0.000261 × *PAEP*. Then, we calculated the TMBPI for SKCM patients and divided them into two TMBPI levels using the median value as the cutoff (**Table S6**). The ROC curve of 3-year OS prediction was drawn to assess the predictive accuracy with AUC = 0.664 (**Figure 7B**). Meanwhile, Kaplan-Meier analysis indicated that SKCM patients with high TMBPI revealed worse survival outcomes compared with low TMBPI, which warranted further investigation and larger samples to validate (**Figure 7C**).

## Discussion

In recent years, there has been a rapid development of immunotherapy for the treatment of advanced melanoma. Nowadays, PD-1 monotherapy is the standard first line therapy for advanced cutaneous melanoma, with efficacy, toxicity, and their correlations well established (30). However, the data from real world showed that durable responses and favorable long-term outcomes are limited to a fraction of patients (31). Thus, many studies are designed to find predictive biomarkers for immune responses.

Indeed, biomarkers remain the major challenge for immunotherapy, not only to identify patients who could respond to treatment but also to avoid unnecessary costs and serve toxicities for non-responders, including hyperprogression under ICB therapy, and to identify candidate patients for combination treatment. TMB and TILs, the novel biomarkers to predict immune responses, had been demonstrated their efficacy in several types of cancer, such as lung cancer, colorectal cancer, cutaneous melanoma, and so on (15, 17, 22, 25, 32). Nevertheless, few relevant researches had focused on the association of TMB with immune infiltrates and their prognostic role in SKCM.

In our study, we investigated the status of TMB in SKCM. The evidence from the analysis of the landscape of mutation profiles in our cohort indicated that 93.36% of patients contain diverse types of mutation. This was consistent to a previous study in which cutaneous melanoma have high mutation load and a predominant C>T nucleotide transition signature attributable to ultraviolet radiation (33). Analysis of correlations between TMB and clinicopathological characteristics showed that higher TMB level correlated with higher age, lower AJCC-T stage, lower AJCC-N stage, and early pathological stage. These data were in accordance with similar results in other clinical trials (NCT01295827 and NCT01866319) that patients with higher age tend to be more sensitive to ICB therapy (34, 35), indicating that TMB is a promising biomarker for response to ICB therapy in SKCM to some extent. Besides, SKCM patients with early pathological stage may be more sensitive to ICB therapy. Inconsistent with previous investigations (26), no significant difference of TMB level was observed with AJCC-M stage in this study. This disparity may be mainly due to unbalanced stages. In our cohort, the frequency of samples with M1 stage was less than 10%.

We also analyzed the relationship between TMB and immune infiltrates in SKCM. We found that most of immune-related genes were downregulated in the lower-TMB group. Meanwhile, the Treg cells were inclined to be upregulated in the lower-TMB group, indicating that high TMB may inhibit immune cell infiltration in the tumor immune microenvironment. Moreover, CNVs of the four hub immune genes were related to immune cell infiltration in SKCM microenvironment. Immune infiltrates including CD8+ T cell, CD4+ T cell, neutrophil cell, dendritic cell, macrophage, and B cell could be inhibited by diverse forms of CNVs carried by hub genes. It implies that TMB have a significant impact on immune cell infiltration, resulting in alteration of tumor immune microenvironment in SKCM. When compared survival prognosis between the higher-TMB group and lower-TMB groups, we found that the higher-TMB group had longer survival time than the lower-TMB group. These data indicated that TMB is correlated to survival prognosis in SKCM, and that the reason leading to this correlation could attribute to the marked differences in immune infiltrates.

In contrast, other researches discovered that the patients in higher-TMB levels are inclined to have poor survival outcomes compared with lower-TMB levels in some types of cancer, including colorectal cancer, clear cell renal cell carcinoma, head and neck squamous carcinoma (26, 29). Seemingly, the association between TMB and survival prognosis exhibited discrepancy in different types of cancer. The disparity may be mainly due to the most of SKCM patients were likely treated with immunotherapy. The data from our cohort and other cancer cohorts implicated that higher-TMB patients could gain a better prognosis than that in lower-TMB patients if treated with immunotherapy, otherwise higher-TMB patients would have poor prognosis compared with lower-TMB patients.

Finally, a prognostic model (TMBPI) was constructed using four hub immune genes which can be used for predicting survival outcomes in SKCM. Patients with high TMBPI revealed poor survival outcomes compared with that with low TMBPI. However, the AUC of the ROC curve was only 0.664 and further large-scale researches are required for verification and modification before clinical application.

There are some limitations, unresolved concerns, and potential perspectives in our study. First, we analyzed the association of TMB with immune infiltrates and their prognostic value using the data from TCGA cohort. The testing cohort from our clinical practice were lacking to evaluate the prognostic role of TMB and its relationship with immune infiltrates. Second, we used CIBERSORT technique to calculate the proportions of immune cells in every patient, which may simply analyze the cell composition on a large scale. However, further flow cytometry and immunohistochemistry are necessary to validate the results. Besides, the genes with “Multi_hit” were not further assessed to figure out which of those genes were the actional mutations. Finally, the basic experiments were lacking to validate the association between four immune genes signature and immune infiltrates.

In summary, we revealed that higher TMB levels correlated with better clinical outcomes and may inhibit immune cell infiltrates in SKCM. Our study also identified four hub TMB-related immune genes, and the CNVs of four hub genes conferred lower immune cells infiltrates. In addition, the prognostic model we constructed indicated that higher TMBPI conferred poor survival outcomes, which warranted further investigation and larger samples to validate.

## Data Availability Statement

The original contributions presented in the study are included in the article/**Supplementary Material**. Further inquiries can be directed to the corresponding author.

## Ethics Statement

The studies involving human participants were reviewed and approved by Medical Ethics Committee of Henan Provincial People’s Hospital. Written informed consent for participation was not required for this study in accordance with the national legislation and the institutional requirements.

## Author Contributions

SC and JuY conceived and designed the study. JuY performed the search and collected the data. XW, JiY, and YZ helped to analyze the results. JuY wrote the manuscript. All authors contributed to the article and approved the submitted version.

## Funding

This work was supported by grants from National Natural Science Foundation of China (82002906, 81902789), Henan Provincial Basic and Frontier Technology Research Project (152300410154), and Beijing Natural Science Foundation (7194244).

## Conflict of Interest

The authors declare that the research was conducted in the absence of any commercial or financial relationships that could be construed as a potential conflict of interest.

## Supplementary Material

Supplementary Figure 1 Summary of the mutation information in SKCM samples. **(A–C)** Statistical calculations of mutation types based on different categories, in which missense mutation accounts for the most fraction, SNP exhibited more frequency than deletion or insertion, and C>T was the most common type of SNV. **(D)** Illustration of tumor mutation burden with the number of variants in each sample. **(E)** Illustration of tumor mutation burden with the median of variants in different mutation types. **(F)** The top 10 mutated genes in SKCM. **(G)** The coincident and exclusive associations across mutated genes. SKCM, skin cutaneous melanoma; SNP, single-nucleotide polymorphism; INS, insertion; DEL, deletion; SNV, single-nucleotide variant.

Supplementary Figure 2 Genecloud plot showed mutation information of genes in SKCM. SKCM, skin cutaneous melanoma.

Supplementary Figure 3 Mutational information of genes in SKCM from cBioPortal for Cancer Genomics. SKCM, skin cutaneous melanoma.

Supplementary Figure 4 Workflow of identifying the hub TMB related immune genes. TMB, tumor mutation burden.

Supplementary Figure 5 Batch survival analysis of four hub TMB related genes in uveal melanoma. TMB, tumor mutation burden.

## References

[1] Fecher LA Cummings SD Keefe MJ Alani RM Toward a molecular classification of melanoma. J Clin Oncol (2007) 25:1606–20. 10.1200/JCO.2006.06.0442 17443002

[2] Siegel RL Miller KD Jemal A Cancer statistics, 2019. CA Cancer J Clin (2019) 69:7–34. 10.3322/caac.21551 30620402

[3] Chen W Zheng R Baade PD Zhang S Zeng H Bray F Cancer statistics in China, 2015. CA Cancer J Clin (2016) 66:115–32. 10.3322/caac.21338 26808342

[4] Curtin JA Fridlyand J Kageshita T Patel HN Busam KJ Kutzner H Distinct sets of genetic alterations in melanoma. N Engl J Med (2005) 353:2135–47. 10.1056/NEJMoa050092 16291983

[5] Curtin JA Busam K Pinkel D Bastian BC Somatic activation of KIT in distinct subtypes of melanoma. J Clin Oncol (2006) 24:4340–6. 10.1200/JCO.2006.06.2984 16908931

[6] Chang AE Karnell LH Menck HR The National Cancer Data Base report on cutaneous and noncutaneous melanoma: a summary of 84,836 cases from the past decade. The American College of Surgeons Commission on Cancer and the American Cancer Society. Cancer (1998) 83:1664–78. 10.1002/(sici)1097-0142(19981015)83:8<1664::aid-cncr23>3.0.co;2-g 9781962

[7] Chi Z Li S Sheng X Si L Cui C Han M Clinical presentation, histology, and prognoses of malignant melanoma in ethnic Chinese: a study of 522 consecutive cases. BMC Cancer (2011) 11:85. 10.1186/1471-2407-11-85 21349197 PMC3056833

[8] Robert C Schachter J Long GV Arance A Grob JJ Mortier L Pembrolizumab versus Ipilimumab in Advanced Melanoma. N Engl J Med (2015) 372:2521–32. 10.1056/NEJMoa1503093 25891173

[9] Postow MA Chesney J Pavlick AC Robert C Grossmann K McDermott D Nivolumab and ipilimumab versus ipilimumab in untreated melanoma. N Engl J Med (2015) 372:2006–17. 10.1056/NEJMoa1414428 PMC5744258 25891304

[10] Zou W Wolchok JD Chen L PD-L1 (B7-H1) and PD-1 pathway blockade for cancer therapy: Mechanisms, response biomarkers, and combinations. Sci Transl Med (2016) 8:328rv4. 10.1126/scitranslmed.aad7118 PMC4859220 26936508

[11] Pentcheva-Hoang T Egen JG Wojnoonski K Allison JP B7-1 and B7-2 selectively recruit CTLA-4 and CD28 to the immunological synapse. Immunity (2004) 21:401–13. 10.1016/j.immuni.2004.06.017 15357951

[12] D’Angelo SP Larkin J Sosman JA Lebbé C Brady B Neyns B Efficacy and Safety of Nivolumab Alone or in Combination With Ipilimumab in Patients With Mucosal Melanoma: A Pooled Analysis. J Clin Oncol (2017) 35:226–35. 10.1200/JCO.2016.67.9258 PMC5559888 28056206

[13] Patel SP Kurzrock R PD-L1 Expression as a Predictive Biomarker in Cancer Immunotherapy. Mol Cancer Ther (2015) 14:847–56. 10.1158/1535-7163.MCT-14-0983 25695955

[14] Dudley JC Lin MT Le DT Eshleman JR Microsatellite Instability as a Biomarker for PD-1 Blockade. Clin Cancer Res (2016) 22:813–20. 10.1158/1078-0432.CCR-15-1678 26880610

[15] Kandoth C McLellan MD Vandin F Ye K Niu B Lu C Mutational landscape and significance across 12 major cancer types. Nature (2013) 502:333–9. 10.1038/nature12634 PMC3927368 24132290

[16] Efremova M Finotello F Rieder D Trajanoski Z Neoantigens Generated by Individual Mutations and Their Role in Cancer Immunity and Immunotherapy. Front Immunol (2017) 8:1679. 10.3389/fimmu.2017.01679 29234329 PMC5712389

[17] Zito Marino F Ascierto PA Rossi G Staibano S Montella M Russo D Are tumor-infiltrating lymphocytes protagonists or background actors in patient selection for cancer immunotherapy. Expert Opin Biol Ther (2017) 17:735–46. 10.1080/14712598.2017.1309387 28318336

[18] Zerdes I Matikas A Bergh J Rassidakis GZ Foukakis T Genetic, transcriptional and post-translational regulation of the programmed death protein ligand 1 in cancer: biology and clinical correlations. Oncogene (2018) 37:4639–61. 10.1038/s41388-018-0303-3 PMC6107481 29765155

[19] Mok T Wu YL Kudaba I Kowalski DM Cho BC Turna HZ Pembrolizumab versus chemotherapy for previously untreated, PD-L1-expressing, locally advanced or metastatic non-small-cell lung cancer (KEYNOTE-042): a randomised, open-label, controlled, phase 3 trial. Lancet (2019) 393:1819–30. 10.1016/S0140-6736(18)32409-7 30955977

[20] Dupuis F Lamant L Gerard E Torossian N Chaltiel L Filleron T Clinical, histological and molecular predictors of metastatic melanoma responses to anti-PD-1 immunotherapy. Br J Cancer (2018) 119:193–9. 10.1038/s41416-018-0168-9 PMC6048096 29973670

[21] Rizvi NA Hellmann MD Snyder A Kvistborg P Makarov V Havel JJ Cancer immunology. Mutational landscape determines sensitivity to PD-1 blockade in non-small cell lung cancer. Science (2015) 348:124–8. 10.1126/science.aaa1348 PMC4993154 25765070

[22] Yarchoan M Hopkins A Jaffee EM Tumor Mutational Burden and Response Rate to PD-1 Inhibition. N Engl J Med (2017) 377:2500–1. 10.1056/NEJMc1713444 PMC6549688 29262275

[23] Ott PA Bang YJ Piha-Paul SA Razak A Bennouna J Soria JC T-Cell-Inflamed Gene-Expression Profile, Programmed Death Ligand 1 Expression, and Tumor Mutational Burden Predict Efficacy in Patients Treated With Pembrolizumab Across 20 Cancers: KEYNOTE-028. J Clin Oncol (2019) 37:318–27. 10.1200/JCO.2018.78.2276 30557521

[24] Horn L Spigel DR Vokes EE Holgado E Ready N Steins M Nivolumab Versus Docetaxel in Previously Treated Patients With Advanced Non-Small-Cell Lung Cancer: Two-Year Outcomes From Two Randomized, Open-Label, Phase III Trials (CheckMate 017 and CheckMate 057). J Clin Oncol (2017) 35:3924–33. 10.1200/JCO.2017.74.3062 PMC6075826 29023213

[25] Lee N Zakka LR Mihm MC Jr Schatton T Tumour-infiltrating lymphocytes in melanoma prognosis and cancer immunotherapy. Pathology (2016) 48:177–87. 10.1016/j.pathol.2015.12.006 27020390

[26] Wang X Li M Correlate tumor mutation burden with immune signatures in human cancers. BMC Immunol (2019) 20:4. 10.1186/s12865-018-0285-5 30634925 PMC6329192

[27] Mayakonda A Lin DC Assenov Y Plass C Koeffler HP Maftools: efficient and comprehensive analysis of somatic variants in cancer. Genome Res (2018) 28:1747–56. 10.1101/gr.239244.118 PMC6211645 30341162

[28] Li T Fan J Wang B Traugh N Chen Q Liu JS TIMER: A Web Server for Comprehensive Analysis of Tumor-Infiltrating Immune Cells. Cancer Res (2017) 77:e108–108e110. 10.1158/0008-5472.CAN-17-0307 29092952 PMC6042652

[29] Zhang C Li Z Qi F Hu X Luo J Exploration of the relationships between tumor mutation burden with immune infiltrates in clear cell renal cell carcinoma. Ann Transl Med (2019) 7:648. 10.21037/atm.2019.10.84 31930049 PMC6944593

[30] Coit DG Thompson JA Albertini MR Barker C Carson WE Contreras C Cutaneous Melanoma, Version 2.2019, NCCN Clinical Practice Guidelines in Oncology. J Natl Compr Canc Netw (2019) 17:367–402. 10.6004/jnccn.2019.0018 30959471

[31] Khozin S Miksad RA Adami J Boyd M Brown NR Gossai A Real-world progression, treatment, and survival outcomes during rapid adoption of immunotherapy for advanced non-small cell lung cancer. Cancer (2019) 125:4019–32. 10.1002/cncr.32383 PMC6899461 31381142

[32] Pan JH Zhou H Cooper L Huang JL Zhu SB Zhao XX LAYN Is a Prognostic Biomarker and Correlated With Immune Infiltrates in Gastric and Colon Cancers. Front Immunol (2019) 10:6. 10.3389/fimmu.2019.00006 30761122 PMC6362421

[33] Hayward NK Wilmott JS Waddell N Johansson PA Field MA Nones K Whole-genome landscapes of major melanoma subtypes. Nature (2017) 545:175–80. 10.1038/nature22071 28467829

[34] Hamid O Robert C Daud A Hodi FS Hwu WJ Kefford R Five-year survival outcomes for patients with advanced melanoma treated with pembrolizumab in KEYNOTE-001. Ann Oncol (2019) 30:582–8. 10.1093/annonc/mdz011 PMC6503622 30715153

[35] Robert C Ribas A Schachter J Arance A Grob JJ Mortier L Pembrolizumab versus ipilimumab in advanced melanoma (KEYNOTE-006): post-hoc 5-year results from an open-label, multicentre, randomised, controlled, phase 3 study. Lancet Oncol (2019) 20:1239–51. 10.1016/S1470-2045(19)30388-2 31345627

