# Supplementary figures and images for: Prognostic Role of Tumor Mutation Burden Combined With Immune Infiltrates in Skin Cutaneous Melanoma Based on Multi-Omics Analysis
Source: Front Oncol. 2020 Nov 10;10:570654. doi: 10.3389/fonc.2020.570654 (PMC7683772; doi:10.3389/fonc.2020.570654)

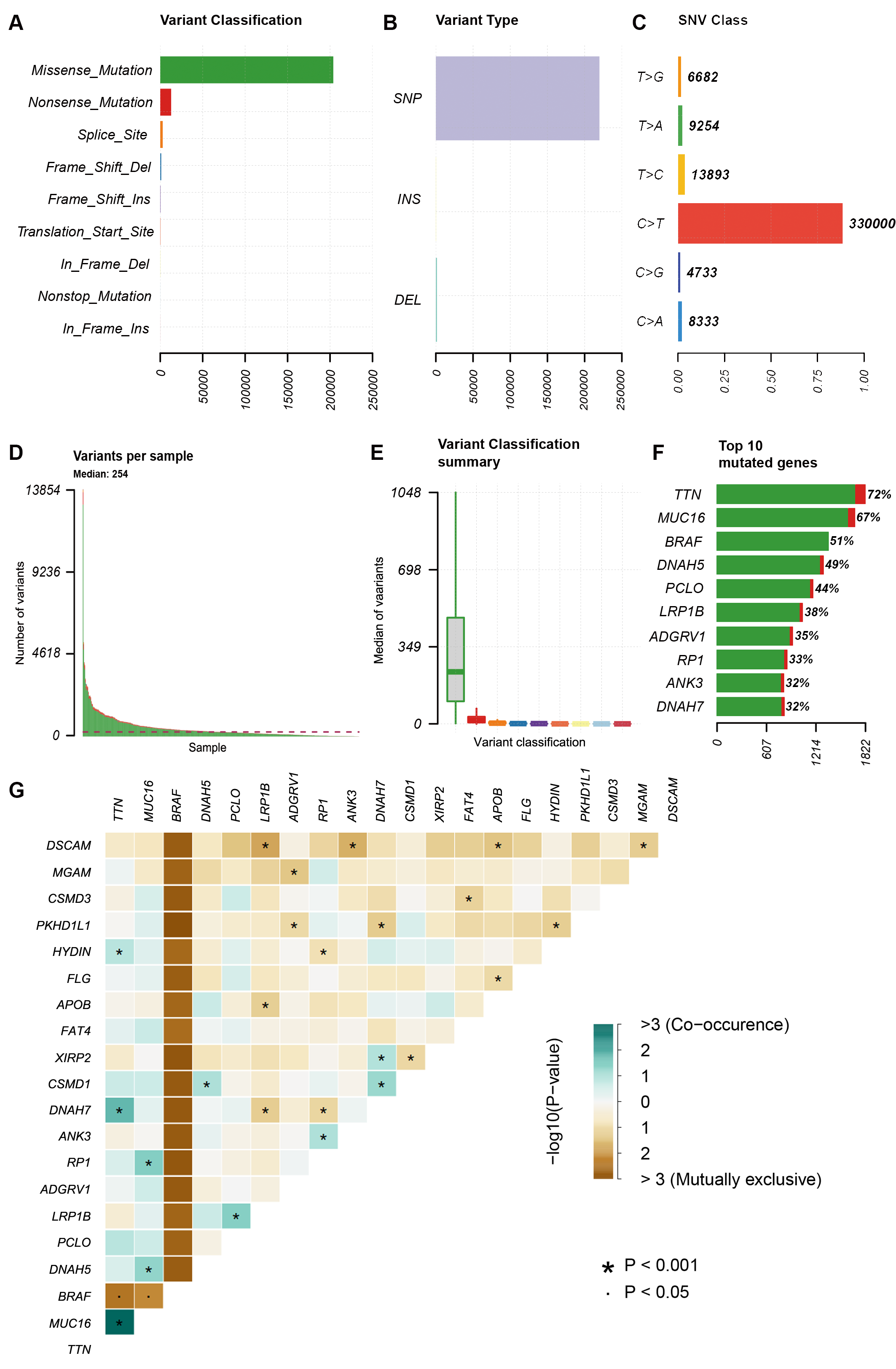

Supplement: Supplementary Figure 1 — Summary of the mutation information in SKCM samples. (A–C) Statistical calculations of mutation types based on different categories, in which missense mutation accounts for the most fraction, SNP exhibited more frequency than deletion or insertion, and C>T was the most common type of SNV. (D) Illustration of tumor mutation burden with the number of variants in each sample. (E) Illustration of tumor mutation burden with the median of variants in different mutation types. (F) The top 10 mutated genes in SKCM. (G) The coincident and exclusive associations across mutated genes. SKCM, skin cutaneous melanoma; SNP, single-nucleotide polymorphism; INS, insertion; DEL, deletion; SNV, single-nucleotide variant. [file Image_1.tif]

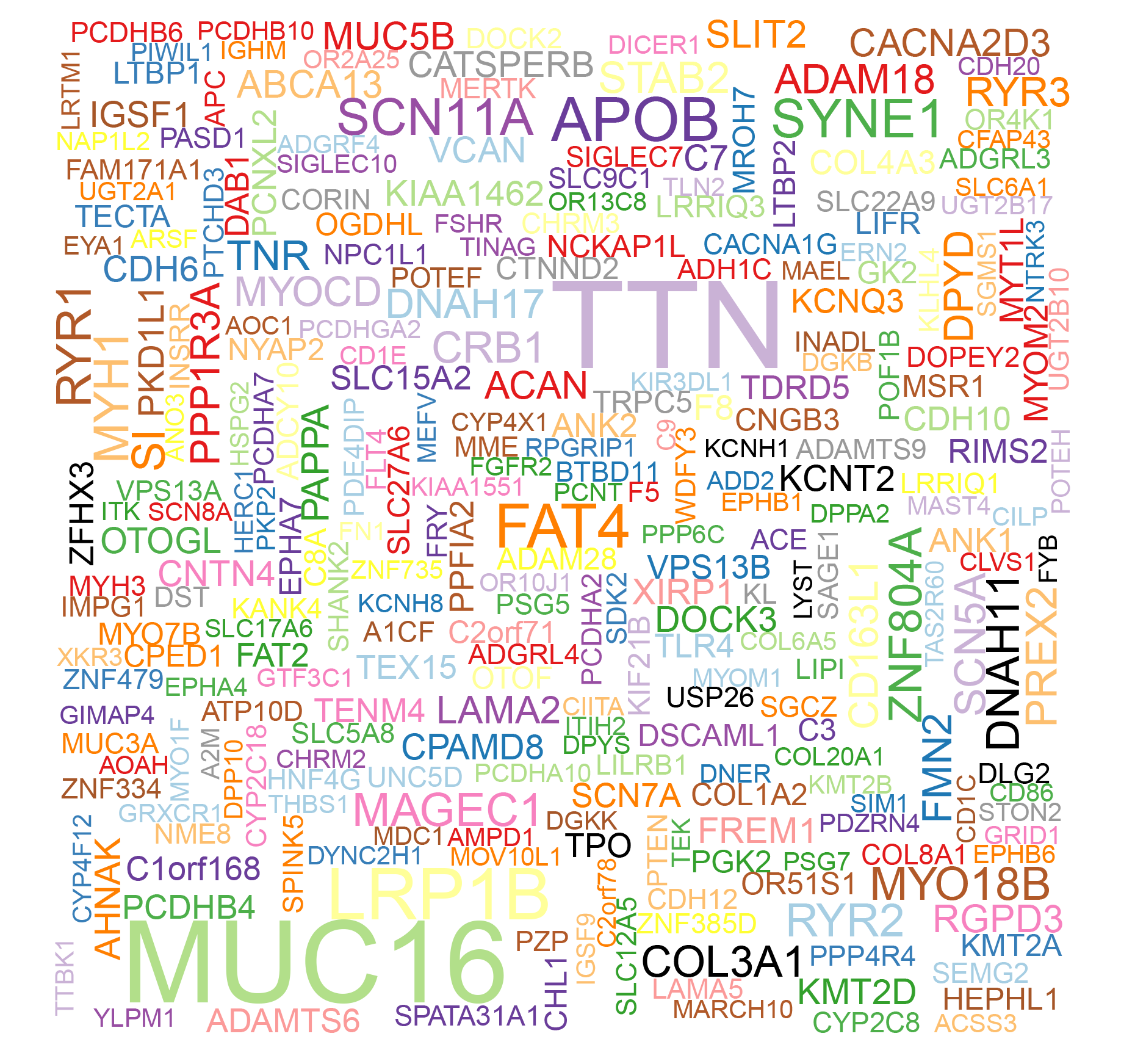

Supplement: Supplementary Figure 2 — Genecloud plot showed mutation information of genes in SKCM. SKCM, skin cutaneous melanoma. [file Image_2.tif]

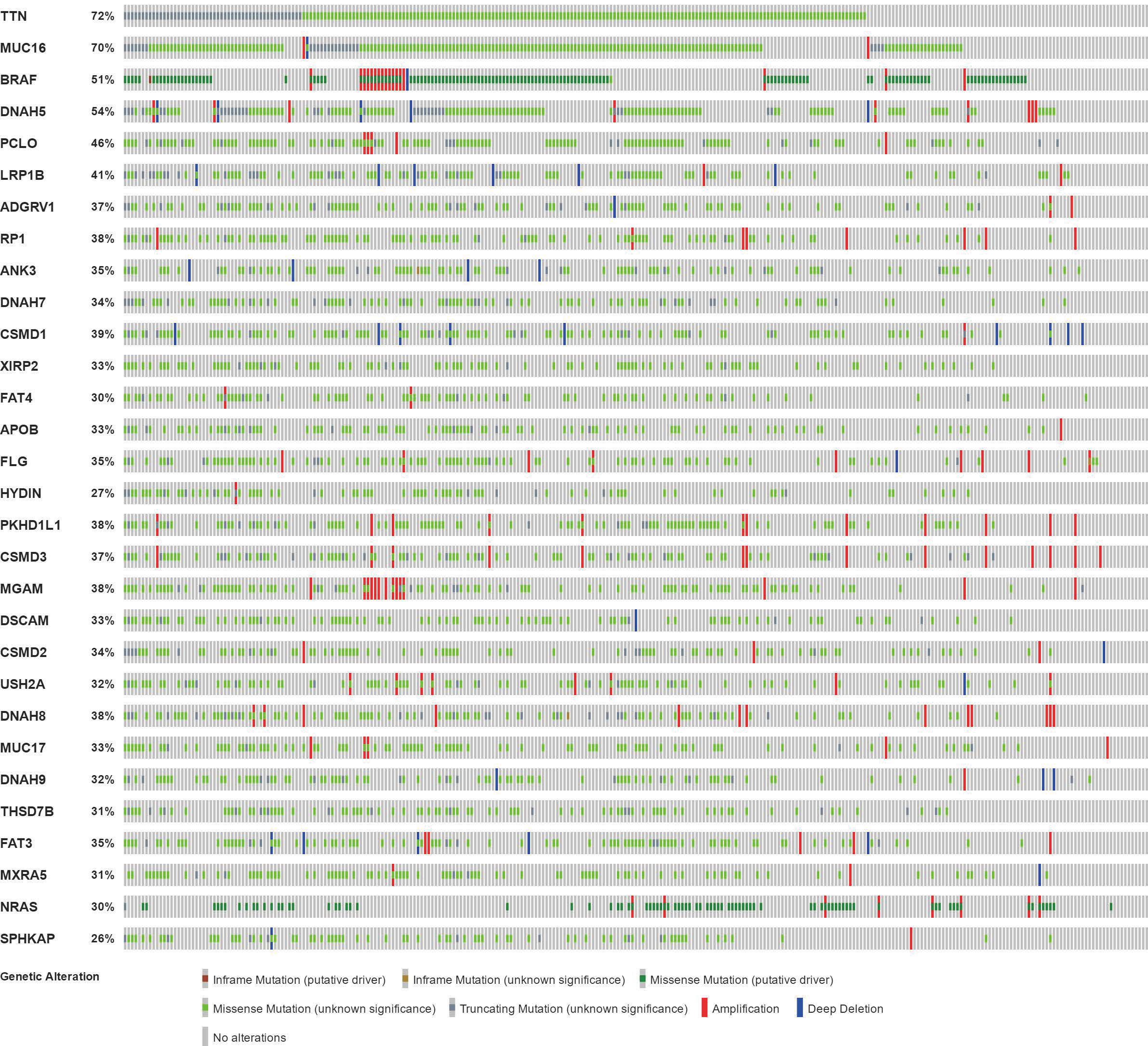

Supplement: Supplementary Figure 3 — Mutational information of genes in SKCM from cBioPortal for Cancer Genomics. SKCM, skin cutaneous melanoma. [file Image_3.tif]

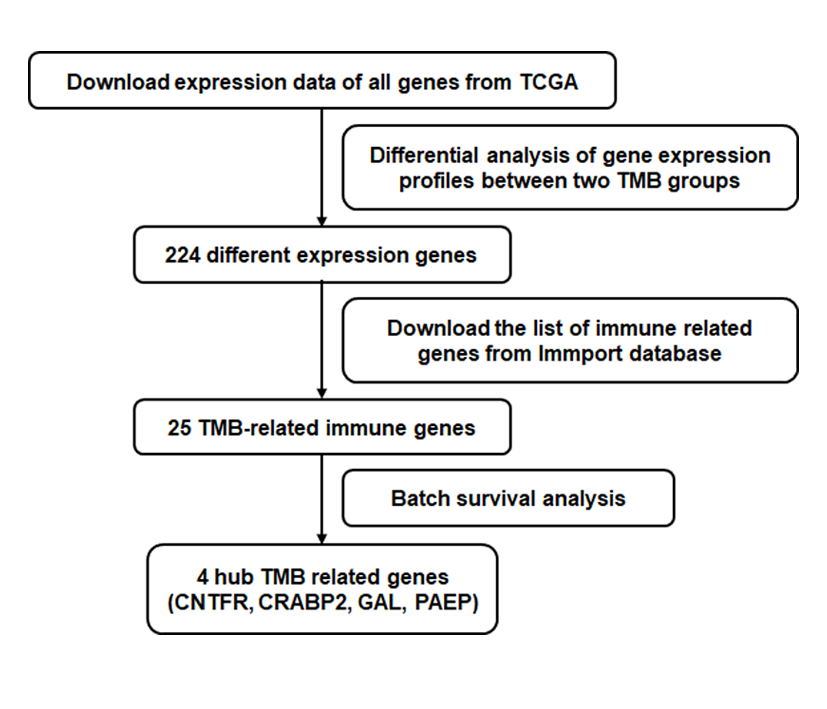

Supplement: Supplementary Figure 4 — Workflow of identifying the hub TMB related immune genes. TMB, tumor mutation burden. [file Image_4.tif]

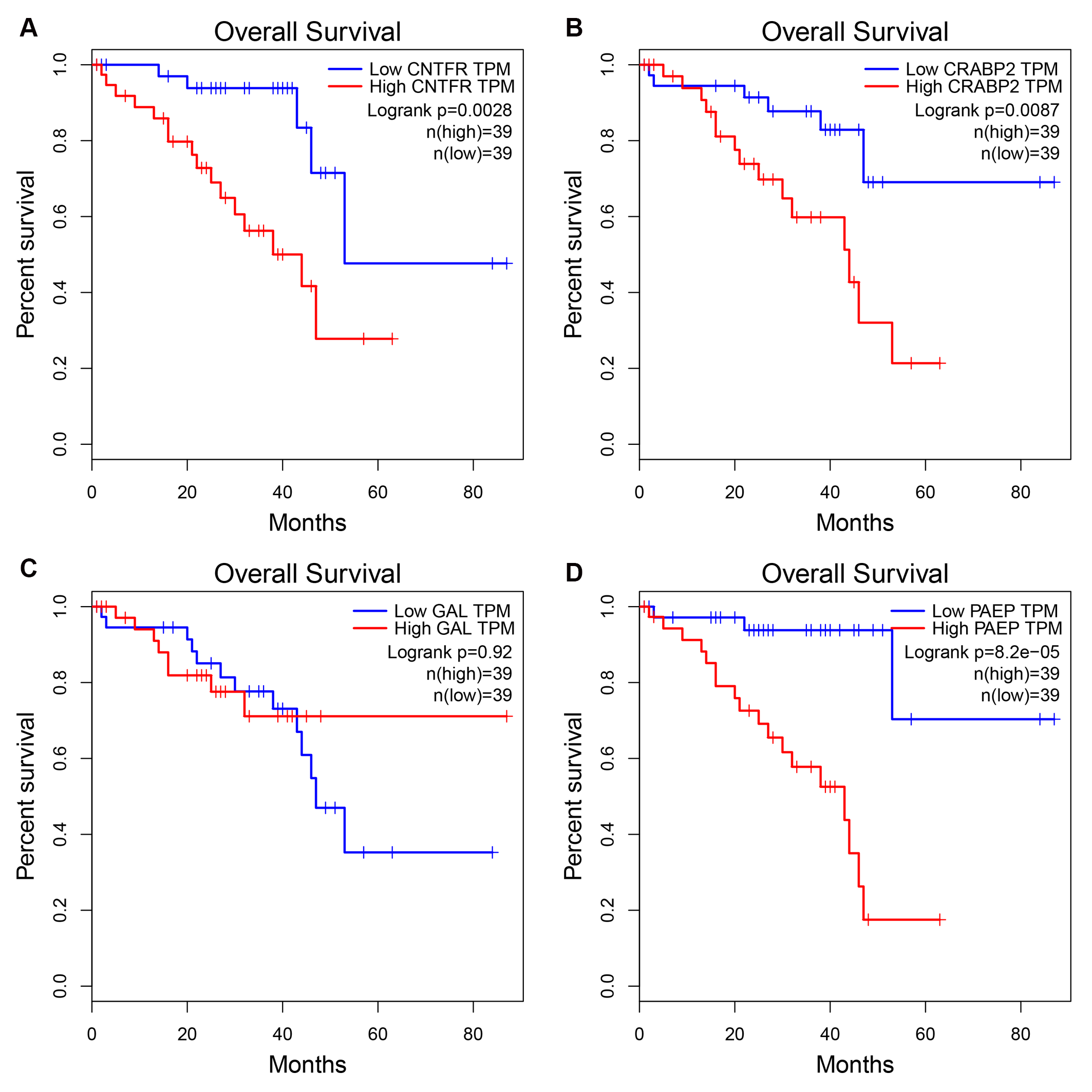

Supplement: Supplementary Figure 5 — Batch survival analysis of four hub TMB related genes in uveal melanoma. TMB, tumor mutation burden. [file Image_5.tif]
